# Association between Physical Functionality and Falls Risk in Community-Living Older Adults

**DOI:** 10.1155/2012/864516

**Published:** 2012-12-04

**Authors:** Disa J. Smee, Judith M. Anson, Gordon S. Waddington, Helen L. Berry

**Affiliations:** ^1^Faculty of Health, University of Canberra, Canberra, ACT 2601, Australia; ^2^Faculty of Applied Science, University of Canberra, Canberra, ACT 2601, Australia; ^3^Centre for Research and Action in Public Health, University of Canberra, Canberra, ACT 2601, Australia

## Abstract

Ageing-related declines in physiological attributes, such as muscle strength, can bring with them an increased risk of falls and subsequently greater risk of losing independence. These declines have substantial impact on an individual's functional ability. However, the precise relationship between falls risk and physical functionality has not been evaluated. The aims of this study were to determine the association between falls risk and physical functionality using objective measures and to create an appropriate model to explain variance in falls risk. Thirty-two independently living adults aged 65–92 years completed the FallScreen, the Continuous-Scale Physical Functional Performance 10 (CS-PFP10) tests, and the 12-Item Short-Form Health Survey (SF-12). The relationships between falls risk, physical functionality, and age were investigated using correlational and multiple hierarchical regression analyses. Overall, total physical functionality accounted for 24% of variance in an individual's falls risk while age explained a further 13%. The *oldest-old* age group had significantly greater falls risk and significantly lower physical functional performance. Mean scores for all measures showed that there were substantial (but not significant) differences between males and females. While increasing age is the strongest single predictor of increasing falls risk, poorer physical functionality was strongly, independently related to greater falls risk.

## 1. Introduction

Every year, 10% of adults aged 75 years and older become dependent because they cannot complete daily activities [1]. Avoiding falls and being physically able to complete tasks necessary for everyday living are essential components of independent living with ageing [2]. One in three community-dwelling adults aged 65 years or older fall each year, and about one-half suffer from multiple falls [3]. Among older adults, falls are the main cause of fractures, hospital admissions for trauma, loss of independence, and injury-related deaths [4]. Falls injuries cause distress, pain, and significant impact on quality of life due to isolation, disability, and a loss of confidence [5]. Falls can also have a financial impact from associated health care costs [6]. Many older adults are afraid of falling [7], and this fear becomes more common as people age, even among those who have not yet fallen. Falling and fear of falling are also potential contributing factors to a decreased mobility and an increased functional dependence [3]. 

Good physical functionality reduces need for care [8], hospitalisation [2], and risk of mortality [9], while declined endurance and altered musculoskeletal integrity and body composition can substantially reduce a person's functional ability or “activities of daily living” [10]. Typical declines in ageing include, but are not limited to, decreases in: muscle strength [11], flexibility [12], balance [13], reaction time [14], and the function of the senses (vision and hearing) [15]. All of these declines bring with them an increased risk of falls [16] and reduced ability to complete daily activities [11] with a consequentially greater risk of losing independence [17].

Most studies have used paper-based methods of falls assessment [18] or physical testing to test ability to complete a small number of everyday tasks (such walking for 6 minutes, getting out of bed, functional reach, climbing stairs, rising from a chair, picking up small objects, and foot tapping) [19], but few have *concurrently *investigated a range of functional tasks. Although these tests can measure individual physical abilities, they fail to measure an individual's overall physical functionality. The FallScreen [20] is a series of objective direct measures of physiological characteristics associated with falls risk, whereas the Continuous-Scale Physical Function Performance 10 (CS-PFP10) [21] is an assessment of functional capacity based on a *combination* of tasks. 

While the risk factors associated with falls are well established [6, 15, 22], as are the age-related declines in physical functionality [18], the relationship between these two has yet to be characterised objectively. The aims of this study were to (i) describe the relationship between falls risk and physical functionality using objective measures and (ii) investigate the relative contribution of age and physical functionality to explain variance in falls risk.

## 2. Methods

### 2.1. Participants

Persons aged 65 years and older, able to speak English able to follow verbal directions, were eligible to participate in the study. Thirty-two individuals residing in an independent-living urban community in Canberra, Australia, volunteered to participate (53% male, 47% female) aged 65–92 years (mean = 77.9, SD = 7.7). Potential participants were contacted via email to attend an information evening about the study and, if willing to participate (*N* = 36), were invited to participate. Following screening, three potential participants were excluded due to chronic disease, and a further one withdrew, leaving 32 participants willing and able to participate. Details of the study were explained prior to commencing with written consent obtained from all individuals. 

### 2.2. Measures

Participants stated their age and sex. Three age categories were created: “*young-old*” (65–74 years), “*old-old*” (75–84 years), and “*oldest-old*” (85 years).

#### 2.2.1. Health

General health was measured using the 12-Item Short-Form Health Survey (SF-12) with the physical (physSF-12) and mental (mentSF-12) subscales identified [23]. Scores for each subscale range from 0 to 100 with higher scores indicating better health. Other health-related data included number of falls in the past 12 months, current medications, and past history of health. 

#### 2.2.2. Physical Functionality

Physical functionality was assessed using the CS-PFP 10, a valid, reliable, and sensitive measure of multiple facets of cardiorespiratory and neuromuscular physiology with no floor or ceiling effects [21] and strongly correlated with self-reported physical functioning measures [24]. It comprises a total score (CS-PFP10 total score) and five physical domains—upper body strength, lower body strength, upper body flexibility, balance, and endurance—each measured by at least two items. These items comprise ten everyday tasks from which outcomes as weight, time, or distance can be measured [24]. Each physical domain can be analysed individually, or they can be combined to provide an accurate global measure of individual functionality. A higher score indicates a higher level of functioning, with no apparent floor or ceiling effects [25].

#### 2.2.3. Falls Risk and Actual Falls

**Falls Risk**

We used the short-form of FallScreen [20], 5-item (vision, peripheral sensation, lower limb strength, reaction time, and body sway) risk calculator that measures five physiological determinants of falls risk. It does not incorporate past falls into the final score. The falls risk score is a single-index score derived from a discriminant function analysis, with a score of less <0 indicating no increased risk of falling. Scores of 0-1 indicate a mild increase in risk, 1-2 moderate risk, 2-3 marked risk, and >3 very marked risk [20]. These assessments are readily accepted by older people, have high external validity and test-retest reliability [20], and are reported to predict those at risk of falling with 75% accuracy in community and institutional settings [22]. Falls risk is presented in “arbitrary units” (AU) [26].

**Reported Falls**

Participants' retrospective self-reported number of falls over the previous 12 months was also recorded.

### 2.3. Statistical Analysis

Using PASW 18 software (SPSS, Inc., 2009, Chicago, IL, USA, http://www.spss.com/), descriptive statistics were produced, and Pearson product-moment correlation coefficients were used to examine bivariate relationships between all variables. Results are presented as means and standard deviations. Multiple hierarchical regression analysis was used to investigate multivariate relationships among predictors of falls risk. In order to improve the application of scientific results to the real world, we chose to employ progressive statistics alongside traditional approaches. To describe the magnitude of the Cohen's effect sizes between physical functioning and falls risk, we used the following descriptors: <0.2 trivial, 0.2–0.6 small, 0.6–1.2 moderate, 1.2–2.0 large, and >2.0 very large [26]. The probability that there was a true change was calculated by accounting for the smallest worthwhile observed difference and typical error of measurement. Thresholds for assigning qualitative terms to these probabilities were as follows: <1% almost certainly not; <5% very unlikely; <25% unlikely; <50% possibly not; >50% possibly; >75% likely; 95% very likely; >99% almost certain.

## 3. Results

Table 1 presents mean scores for age, falls risk, and physical functional performance total scores (CS-PFP10 total score) including the five domain scores and the scores from the SF12 including the physical (SF12phys) and mental (SF12men) components. Substantial (but not always significant) differences were evident between males and females, with trends in mean scores for upper body flexibility, and lower body flexibility, upper body strength and the physical component scores in the expected directions: females recorded significantly greater upper body flexibility than males did, whereas males had greater lower body strength and physical functioning, with nearly significantly greater upper body strength (*P*  value = .06). Four males and four females reported at least one fall within the past 12 months (25% of the sample, consistent with national prevalence [27]) with two females reporting multiple falls.

Age-group mean scores differed significantly for falls risk, upper body strength, lower body strength, balance, and endurance. Those in the *young-old group* had significantly higher mean scores (better functioning) than did the *oldest-old group* in the following areas: physical functional performance total score, upper body strength, lower body strength, balance, and endurance. Those in the *young-old group* had significantly lower mean scores than did the *oldest-old group* in falls risk scores. Those in the *old-old group* had significantly lower falls risk than did the *oldest-old group* for falls risk only, meaning that they were less likely to fall than were the *oldest-old group*. Mean scores for the physical and mental components did not differ significantly between the age groups.

Associations among measures of sex, age, falls risk, physical functional performance, and health are shown (Table 2). Significant associations (all moderate to strong) were apparent between: (i) age and falls risk, with younger participants showing a lower falls risk; (ii) age and physical functional performance total score and the associated domain scores (upper body strength, lower body strength, upper body flexibility, balance, and endurance), with younger people showing better functionality. Domain scores of the Continuous-Scale Physical Functional Performance were significantly positively correlated with physical functional total score because they contribute to the total score. There was no association between age and general health (SF12), but there was a strong relationship between the physical component score of the SF12, total physical function, and all five domains such that better functioning on one was associated with better functioning on all the others. There was no association between reported falls and falls risk. In addition, there was no statistical difference between the CS-PFP10 total score of fallers compared to nonfallers.

A multiple linear regression (Table 3) was undertaken to estimate the contribution of (i) physical functioning and (ii) age to explain variance in falls risk. The two assessment tools used (FallScreen and CS-PFP 10) are correlated (*r* = −.492, *P* < .004) but not strongly, that is, the two measures are *not* collinear and *do not* measure the same thing. Total physical functionality accounted for 24% of variance in falls risk with age contributing a further 13%. Using progressive statistics, there is a 75% probability that physical functionality performance is a true component of the model for falls risk. A multiple linear regression analysis using the five components of the CS-PFP10 to predict falls risk showed that these physical domains were intercorrelated; only “endurance” made a significant independent contribution to explain the variance in falls risk.

Unstandardised Beta values obtained from the final regression model (physical functionality controlling for age) were used to estimate age-related falls risk and risk category (Table 4). The table shows how falls risk can be estimated by an individual's age.

## 4. Discussion

This study is the first to investigate the relationship between falls risk and physical functionality using two objective and validated assessment tools. Although there were no significant differences between women's and men's physical functionality, men tended to have a greater overall strength and physical functioning, while women tended towards, having a greater upper body flexibility. These findings are consistent with other studies which have found significant gender differences in functional tasks [28]. The “gender gap,” with women being less physically able than men, is markedly increased with age. Age-related declines generally were also evident. The *oldest-old* group was more likely to fall compared to both of the younger groups. This is consistent with other studies finding, that is, increasing age is accompanied by increasing risk of falling [29]. The decline from the *young-old* to *oldest-old* groups was marked. Not surprisingly, the *oldest-old* group was less physically able and was weaker in both of their upper and lower body. They also had poorer balance and poorer endurance as indicated in Table 1. There was evidence of a dose-response effect in age-related decline. That is, people in the *oldest-old* group were less functional than the *old-old*, who were, in turn, less functional than the *young-old* group. The declines in CS-PFP10 task specific functionalities help to explain previously observed deteriorations in overall ability to undertake activities in daily living [28]. 

Our findings are consistent with other studies that have demonstrated that, throughout ageing, individuals develop progressively poorer upper and lower body strength and endurance and greater risk of falling, with age-related declines in physical functionality contributing to falls risk [28, 30]. This is the first study to demonstrate this pattern of results using all three measures, simultaneously. Other studies have previously shown a relationship between physical capability and falls [30], but none has associated falls risk with specific functional tasks. The present study shows that (i) objectively measured components of absolute strength (FallScreen subcomponents) and (ii) measures of functional capacity related to strength (CS-PFP10 physical domain) overlap each other but contribute separately to understanding falls risk. 

We note that we found no association between reported falls and falls risk. This is inconsistent with the findings of other studies [15] and most likely due to our very small sample size.

Because self-reported scores on the physical component of the SF12 measure were strongly positively related to physical functionality and all associated domains, we can infer that individuals are capable of realistically assessing their own level of physical functioning. However, we found no relationship between self-reported physical functioning and the risk of falls, potentially indicating that the self-reported survey is not sensitive enough to elucidate impending falls.

We have used the results from this study to predict age-related falls risk (Table 4), which could prove a valuable tool for clinicians in providing evidence about falls to their older clients.

There are two main limitations of this study. Firstly, the number of the outcome results did not attain statistical significance, most likely due to the small sample size, but the results were never less consistent with previous findings. The present preliminary study provides information on which power calculations for specifying sample size requirements in future studies will be based. Secondly, although it is well known that health can be influenced by social factors [31], we had little socioeconomic data to assist in accounting for unexplained variance in our model predicting falls risk. We also had limited demographic information thus; evidence pertaining to social connectedness could not be incorporated into this study. Future studies would be improved by including health and life-style measures, such as physical activity level, smoking, diet, alcohol consumption, and social connectedness. In addition, it would be helpful to include a “fear of falling” questionnaire to improve understanding and interrelationships of falls and falls risk. 

Despite these limitations, this study provides evidence for using physical functionality and age to predict falls risk in community-living older adults. Using objective measures, we have shown a strong relationship between falls risk and physical functionality. We have also shown how ageing *and* falls risk are interdependently and independently associated with a number of physical impairment factors, such as reduced balance and muscle weakness. 

As individuals age, their falls risk increases. Falls can have a devastating effect on independence and quality of life, often leading to a spiral of inactivity and even further decline in functionality, increased falls risk, and greater likelihood of requiring assisted living. Using concurrent testing for falls risk and physical functionality, we showed the negative relationship between the two, but also that age is a critical determinant of falls risk. Importantly, we provide a simple, clinically useful “ready-reckoner” for service providers working with older people which could be used to help to plan individual interventions to reduce falls risk. Encouraging older people to improve their physical functionality could help them to retain their independence and reduce the need for assisted living.

## Figures and Tables

**Table 1:** Sex and age-group differences in falls risk, physical functional performance, and health for sample participants (aged 65–92 years).

	Sex		Age-groups		
	Female (*n* = 17)	Male (*n* = 15)	65–74 (*n* = 10)	75–84 (*n* = 16)	>85 (*n* = 6)
Age (years)	76.8 ± 7.50	79.3 ± 8.01	68.8 ± 2.73	79.6 ± 3.26	88.8 ± 7.70
Actual falls (12 months)	4	4	2	5	1
Falls risk (AU)	1.28 ± 1.06	1.29 ± 1.42	0.79 ± 0.82	1.05 ± 0.99	2.76 ± 1.33^∗a^
CS-PFP10 total score (AU)	44.5 ± 14.15	46.8 ± 16.06	56.3 ± 11.29	44.0 ± 13.66^#b^	32.0 ± 11.05^∗c^
Upper body strength (AU)	38.2 ± 13.78	49.3 ± 18.41^#d^	53.2 ± 14.60	41.8 ± 15.22	31.3 ± 16.94^∗c^
Lower Body strength (AU)	37.9 ± 18.05	42.0 ± 16.83	49.9 ± 12.52	38.9 ± 17.80	25.5 ± 12.81^∗c^
Upper body flexibility (AU)	59.2 ± 15.05	52.0 ± 12.72	63.8 ± 11.53	53.8 ± 15.04	48.0 ± 10.96^#b^
Balance (AU)	47.6 ± 14.46	47.4 ± 16.78	58.6 ± 11.62	46.0 ± 14.11^#b^	32.9 ± 9.85^∗c^
Endurance (AU)	46.9 ± 14.61	47.7 ± 16.42	58.5 ± 11.25	45.7 ± 14.11^#b^	32.8 ± 10.26^∗c^
SF12phys (AU)	43.8 ± 12.12	47.3 ± 6.65	50.1 ± 9.74	43.4 ± 10.22	45.4 ± 9.90
SF12men (AU)	52.7 ± 9.32	53.8 ± 7.01	53.4 ± 7.31	53.3 ± 9.22	52.9 ± 8.14

^#^ *P *value < 0.10, *P value < 0.01.

^a^Mean score is significantly higher (greater risk of falls) than for the young-old and old-old groups.

^b^Mean score nears being significantly lower (worse) than the young-old group.

^c^Mean score is significantly lower (worse) than for the young-old group.

^d^Mean score nears being significantly higher (greater strength) than women.

**Table 2:** Correlation analysis between, sex, age, falls risk, physical functional performance, and health for sample participants (aged 65–92 years) (italic data are the domain score physical functional performance).

	2	3	4	5	i	ii	iii	iv	v	Falls risk
(1) Age (years)	0.17	−0.28	−0.04	−0.61**	−0.45**	−0.52**	−0.44*	−0.63**	−0.62**	0.59**
(2) Sex		0.18	0.07	0.08	0.34^#^	0.12	−0.26	−0.01	0.03	0.01
(3) SF12phys (AU)			−0.08	0.53**	0.51**	0.56**	0.47**	0.49**	0.52**	−0.17
(4) SF12men (AU)				0.04	−0.03	0.04	0.11	0.05	0.04	0.04
(5) CS-PFP10 total score (AU)					0.86**	0.94**	0.74**	0.99**	0.53**	0.49**
(i) Upper body strength (AU)						0.83 **	0.44 *	0.77 **	0.79 **	0.40 *
(ii) Lower body strength (AU)							0.72 **	0.92 **	0.92 *	0.41 *
(iii) Upper body flexibility (AU)								0.78 **	0.76 **	*0.29 *
(iv) Balance (AU)									0.99 **	−0.51 **
(v) Endurance (AU)										−0.51 **

^#^ *P* value < 0.10, *P value < 0.05, and **P value < 0.01.

**Table 3:** Multiple linear regression model predicting falls risk.

Predictor	Unstandardised *β*	SE B	Standardised Beta	*R* ^2^ change
Model 1				
CS-PFP10 total score	−0.04	0.01	−0.49**	0.24
Model 2				
CS-PFP10 total score	−0.02	0.02	−0.23	
Age	0.07	0.03	0.46	0.13*

*P value < 0.05, **P value < 0.01.

**Table 4:** Estimates of age-related falls risk (based on age and physical functionality).

Age (years)	Estimated falls risk (AU)	Falls risk category
65	0.08	Low-Mild
70	0.54	Mild
75	1.0	Moderate
80	1.5	Moderate
85	1.9	Moderate-Marked
90	2.4	Marked
